# Molecular Analysis of *Aquaglyceroporin 1* Gene in Non-Healing Clinical Isolates Obtained from Patients with Cutaneous Leishmaniasis from Central of Iran

**Published:** 2019-06-24

**Authors:** Yasaman Alijani, Saeedeh Sadat Hosseini, Salman Ahmadian, Sonia Boughattas, Gilda Eslami, Shadi Naderian, Vahid Ajamein

**Affiliations:** 1 Research Center for Food Safety and Health, Shahid Sadoughi University of Medical Sciences and Health Services, Yazd, Iran; 2 Department of Parasitology and Mycology, School of Medicine, Shahid Sadoughi University of Medical Sciences and Health Services, Yazd, Iran; 3 Biomedical Research Center, Qatar University, Doha, Qatar; 4 Department of Statistics and Epidemiology, School of Public Health, Shahid Sadoughi University of Medical Sciences and Health Services, Yazd, Iran

**Keywords:** Leishmaniasis, Cutaneous, Drug rsesistance, Antimony

## Abstract

**Background:**

Regarding the antimonial-resistant of *Leishmania* spp., understanding of related mechanism is necessary. One of the most important involved molecules is aquaglyceropin1 (AQP1). The aim of this study was molecular analysis of *AQP1* gene from antimonial-resistant clinical isolates and its expression.

**Methods:**

Overall, 150 patients with cutaneous leishmaniasis referring to the reference laboratories of Yazd and Varzaneh,, located 105km southeast of Isfahan and 240km away from Yazd, were assessed from Jun 2015 to Dec 2017. After sampling, staining was done and evaluated for Leishman by microscope. Samples were collected in RNAlater solution for gene expression analysis in non-healing isolates. DNA extraction was performed from each slide with Leishman body. All patients with *L*. *major* isolates detected by ITS1-PCR-RFLP were followed for finding the resistant isolates, consequence of molecular characterization of *AQP1* using PCR-RFLP. Gene expression of *AQP1* from all resistant isolates was assessed in comparison with the one in a sensitive isolate. Statistical analysis was done using SPSS. The significance level was considered ≤0.05.

**Results:**

Five isolates were detected as antimonial resistant. Molecular detection and identification were appeared that all were *L. major*. The molecular characterization of *AQP1* showed G562A mutation. Gene expression of *AQP1* in resistant isolates showed 1.67 fold higher than the sensitive isolate.

**Conclusion:**

We reported a new point mutation of G562A in *AQP1* gene involved in molecular mechanism in resistant isolates.

## Introduction

Leishmaniasis, caused by *Leishmania* spp., is one of the most important parasitic diseases which occurs in various forms including cutaneous (CL), muco-cutaneous, disseminated cutaneous, and visceral. CL is resulted from *L. major* and *L. tropica* in the Old World. The first line of treatment for CL is pentavalent antimony [Sb(V)] such as sodium stibogluconate (pentostam®) and meglumine antimonite (glucantime®) (1). One of the important clinical concerns for the treatment of various infectious diseases especially CL is drug resistance. Resistance to antimonial drugs as the first line of treatment has been reported in leishmaniasis in several countries (2–4). One of the important molecules involving antimonial resistance is Aquaglyceroporin 1. This protein that encoded from *AQP1* gene presented on chromosome 31 has critical role in transporting the antinomial drug inside the parasite and therefore any changes in its expression and gene sequence may involve in antimonial resistance development (5, 6).

Based on our knowledge, the presence of a point nucleotide mutation in the genes involved in drug absorption comes into effect by interacting with the drug and the target or changing the permeability of the drug into the parasite (7). Three antimonial-resistant isolates had a deletion of 64 of 204kb base pairs at the end region of chromosome 31 (8). This deletion is included parts of *AQP1* gene. Another study found a nucleotide change in position of 398 in *AQP1* gene from non-healing isolates that resulted in changing the amino acid glycine to aspartic acid that it seemed to affect the absorbance of antimonial (9). Recently, we reported a new mutation of G562A in *AQP1* gene from the standard isolate of *L. major*.

For finding any correlation of this mutation and molecular mechanism of antimonial resistance, in this study, we investigated the molecular characterization and gene expression of *AQP1* in resistant isolates obtained from patients with CL.

## Materials and Methods

### Population study

In this cross-sectional study, the research population included suspected cases of CL referring to the reference laboratories of two different endemic regions of Iran, including Yazd, located 270km (170mi) southeast of Isfahan, and Varzaneh, located 105km southeast of Isfahan and 240km away from Yazd, from Jun 2015 to Dec 2017.

Totally, 150 patients were considered for assessing. All patients were followed at least for 3 months after treatment to finding the resistant isolates.

The informed consent was completed from all patients included in this study. The ethical approval with the code of IR.SSU. MEDICINE.REC.1394.532 has been taken from the Research Ethics Committee of Shahid Sadoughi University of Medical Sciences, Yazd, Iran.

### Sampling

After sterilizing, the edge of each lesion was sampled and placed on two separate slides. After preparing smear, it was fixed using methanol. The Giemsa staining was done. Then, Leishman body was screened using optical microscope. All positive samples were considered for the next steps. Moreover, sample of each patient was collected in RNAlater solution for gene expression of AQP1 in the non-healing clinical isolates in next steps.

### DNA extraction

DNA extraction was done using DNA extraction kits (GeneAll, Tissue, and blood, Exgene cell SV, Korea, Seoul, #106-101) from the slides containing the parasite. The protocol was performed based on the company instruction by little modification. Each slide was washed by xylol. After drying, the samples of each slide scratched and transferred into a sterile 1.5ml microtube. The other steps were done based on the kit instructions.

### Detection and identification of species

Since the target population was *L. major* isolates, the ITS1-PCR-RFLP was used to detection and identification (10) using the specific primer pair of LITSR: 5′-CTGGATCATTTTCCGATG-3′ and L5.8S: 5′-TGATACCACTTATCGCACTT-3′. The amplification was done using thermocycler (ABI-SimpiAmp, USA). The presence of a fragment of 300–320bp in length detected *Leishmania* spp. For identification, RFLP was done using *Hae* III restriction enzyme. The amplification and digestion analysis were done by agarose gel electrophoresis alongside by 50bp DNA ladder. Fragments of 200 and 127bp in length after enzyme digestion, was considered as *L. major*. All patients with *L. major* isolates were followed for three month for finding the resistant ones.

### Molecular analysis of AQP1

The non-healing clinical *L. major* isolates were included for G562A mutation analysis. The specific primer pair of LmAQP1-F: 5′-TGTCTGGTGGTCACACTTGAAC-3′ and LmAQP1-R: 5′-CACGACTAGAGGTATCCAAAAGTA-3′ was used for *AQP1* gene amplification. The amplification was done using thermocycler (ABI-SimpliAmp, USA). The reaction was done in a volume of 20µl containing 10pmol each primer, 1U Taq DNA polymerase, 1X PCR buffer, 1.5mM MgCl_2_, 0.2mM dNTP, and 100mg DNA as template. The reaction program included the first denaturation of 94 °C for 5min, followed by 40 cycles of 94 °C for 1min, 56 °C for 1min, and 72 °C for 1min. The final extension was done at 72 °C for 5min. The amplicon size of 509bp in length showed the *AQP1* amplification. Then, RFLP analysis was done with *Zra* I resctriction enzyme for G562A assessing. The pattern with the fragments of 198 and 311bp in length was considered as wild type and no mutation but no digestion showed G562A mutation. Amplification and digestion analyses were done using agarose gel electrophoresis (1%) alongside with 50bp DNA ladder.

### Gene expression

Total RNA from the non-healing clinical isolates was extracted using the GF-1 Total RNA Extraction Kit (Vivantis, Malaysia, #GF-TR-025). The quality and quantity of the extracted RNA were done using 1% agarose gel electrophoresis and spectrophotometer (Eppendorf BioPhotometer plus, Eppendorf, Germany), respectively. The cDNA synthesis was performed using RevertAid™ First Strand cDNA Synthesis Kit (Thermo Fisher Scientific, USA, K1621) based the protocol. All antimonial-resistant clinical isolates were assessed in order to *AQP1* gene expression using the specific primer pair of AQP1-F: 5′-AGTGTGGAGCGAGGTGTTCAA-3′ and AQP1-R: 5′-CCGAGAGTATGCGAGGTGACAA-3′ (2). A susceptible strain was considered as the standard one. The Glyceraldehyde 3-phosphate dehydrogenase (GAPDH) gene was evaluated as the endogenous control with the specific primer pair of GAPDH-F: 5′-CCGAGAGTATGCGAGGTGACAA-3′ and GAPDH-R: 5′-GCCCCACTCGTTGT CATACCA-3′. The amplification was done with thermocycler (Applied Biosystem step one; USA). The amplification was done in triplicate. The Real Time PCR program comprises the initial denaturation at 95 °C for 10 min followed by 40 cycles of denaturation at 95 °C for 10sec and annealing/extension at 60 °C for 20sec. The specificity of amplification was performed by melting curves analysis.

The analysis was performed using ΔΔCt as (ΔCt_sample-target_−ΔCt_sample-control_)−(ΔCt_standard-target_−ΔCt_standard-control_) and the Relative Quantitative (RQ) was calculated by 2^−ΔΔCt^.

### Statistical analysis

Data were analyzed using SPSS-16 software (Chicago, IL, USA). Chi-square test was used to compare qualitative variables. The significance level in the applied tests was ≤ 0.05.

## Results

Five age groups were classified. We classified the ages from 0 to 13 as group 1, 14 to 20 as group 2, 21 to 40 as group 3, 41 to 50 as group 4, and over than 50yr old as group 5. Totally, 41 from group 1, 19 from group 2, 65 from group 3, 18 from group 4, and 7 from group 5 were included in this study. All isolates from groups 1 and 2 were susceptible to treatment, while out of the isolates from group 3, three were non-healing and 62 were susceptible to treatment. Among the isolates from group 4, one was non-healing and 17 were sensitive to treatment. Among the group 5, one was non-healing and six were susceptible to treatment. Thus, the amount of K-square was measured as 5/28 with a significant level of P= 0.260, and therefore the response to therapy and age group did not correlate with each other and act independently (Table 1).

**Table 1.** Correlation of the drug response and age category

			**:answer Treatment**		**Total**		

			**Non-healing**	**Sensitive**			

						**Chi square**	**P**
**ID**	Group 1 (0–13)	Count	0	41	41	5.286	0.260
		% within answer Treatment	0.0%	28.3%	27.3%		
	Group 2 (14–20)	Count	0	19	19		
		% within answer Treatment	0.0%	13.1%	12.7%		
	Group 3 (21–40)	Count	3	62	65		
		% within answer Treatment	60.0%	42.8%	43.3%		
	Group 4 (41–50)	Count	1	17	18		
		% within answer Treatment	20.0%	11.7%	12.0%		
	Group 5 (over than 50 years old)	Count	1	6	7		
		% within answer Treatment	20.0%	4.1%	4.7%		

**Total**		Count	5	145	150		
		% within answer Treatment	100.0%	100.0%	100.0%		

Out of 150 patients with CL, 84 were men and 66 were women. Out of 84 men, just one patient was resistant to treatment with antimony and others were treated immediately after first prescription. Out of 66 women, four did not cure after three months considered as resistant to treatment. Totally, 5 isolates were non-healing. All non-healing isolates were regarding to Varzaneh. All isolates obtained from the patients with CL from Yazd were susceptible to antimonial drug.

**Fig. 1.**
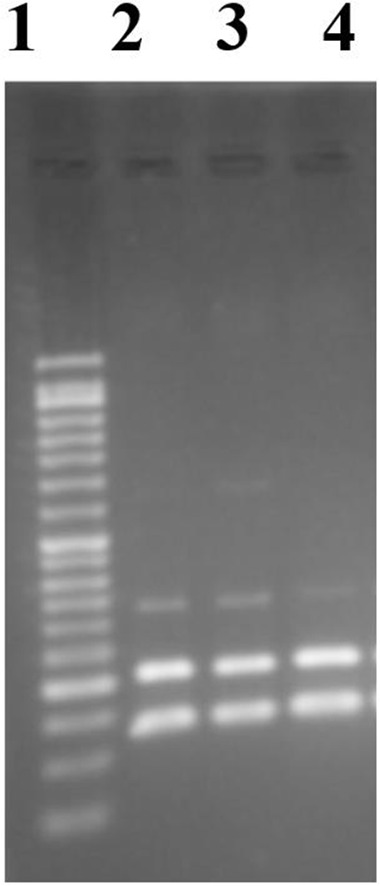
Agarose gel electrophoresis for detection and identification of clinical isolates. ITS1-PCR-RFLP with the result of fragments with 100 and 200bp in length showed *Leishmania major.* Lane 1: 50bp DNA ladder, lane 2: standard *L. major*, lanes 3, 4: clinical isolates with the pattern of *L. major* after restriction analysis

ITS1-RFLP-PCR was done for isolates. The results of PCR from the antimonial-resistant clinical isolates showed an amplicon of around 3bp in length. Then, *Hae* III restriction enzyme analysis showed *L. major* pattern with the fragments of 200 and 100bp in length (Fig. 1).

**Fig. 2.**
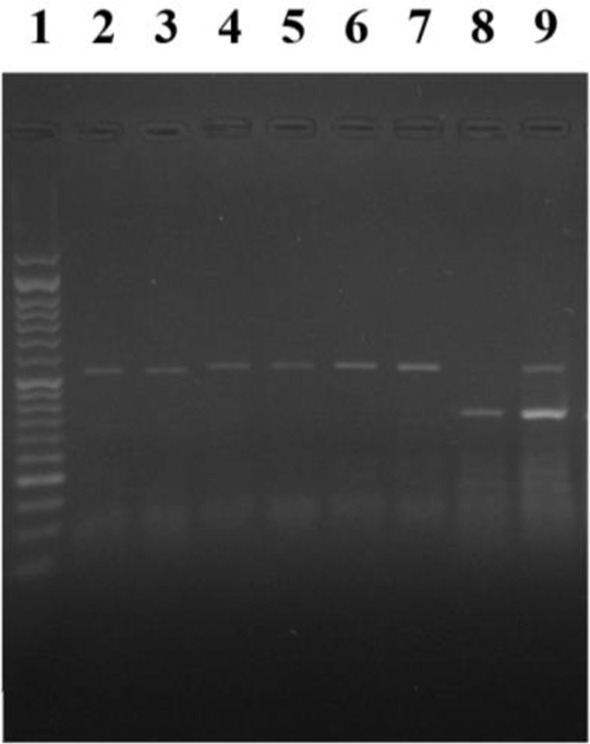
Agarose gele electrophoresis for PCR-RFLP analysis for *AQP1* gene in antimonial-resistant clinical isolates. Lane 1: 50bp DNA ladder, lane 2: the fragment size of 509bp in length in positive control of *Leishmania major* after PCR-RFLP, no digestion showed G562A mutation; lanes 3–7: the fragment size of 509bp in length in five antimonial-resistant clinical isolates of *L. major* after PCR-RFLP, no digestion showed G562A mutation, lane 8: the fragment sizes of 198 and 311bp in length susceptible *L. major* isolate after PCR-RFLP

Molecular analysis of *AQP1* sequencing for the mentioned mutation analysis showed all five isolates had G562A (Fig. 2).

**Fig. 3.**
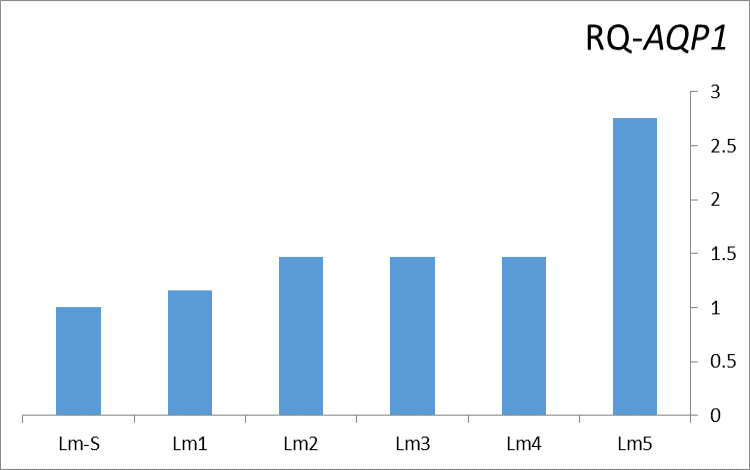
Relative quantitative (RQ) of *LmAQP1* in antimonial resistance clinical isolates of *L. major* by Real-Time PCR. Lm-S: The standard isolate, Lm1 to Lm5: the antimonial drug resistance clinical isolates

The mean RQ of *AQP1* from the non-healing clinical isolates showed 1.67 fold higher than the standard isolate as the sensitive one (Fig. 3).

## Discussion

We reported here the molecular characterization of non-healing *L. major* clinical isolates, including G562A mutation in *LmAQP1* and higher gene expression of *LmAQP1* in comparison with the susceptible isolates. Moreover, the age and sex of the patients had not any correlation with non-healing isolates. *AQP1* encodes the aquaglyceropin protein which is one of the important factors in antimony resistant isolates (11). LmjAQP1 has critical roles in the parasite, including transporting solute such as water, glycerol, methylglyoxal, dihydroxyacetone, and sugar alcohols, regulating the volume, osmotaxis (12), and especially up taking of Sb (III). In this study, the mutation of G562A in *AQP1* makes the alteration of alanine to threonine. The gene expression of *LmAQP1* in all non-healing isolates was 1.67 fold more than the one in susceptible one. This mutation has especial role for increasing the *LmAQP1* gene expression in this isolates.

Gene expression regulation in *Leishmania* is controlled post-transcriptionally (13). Mitogen-Activated Protein Kinase (MPK) is the one of important key for control of *AQP1* gene expression (14). Threonine is one of the important amino acid that could be the target of protein kinase for phosphorylation. The kinases normally phosphorylated the OH group of threonine. In isolates studied in this study, G562A mutation was reported with substitution of alanine by threonine. More phosphorylation of *LmAQP1* make overexpression of the gene (14). Phosphorylation of threonine in LmAQP1 stabilizes the protein and therefore increasing of the half-life of the AQP1 makes it overexpression. This overexpression resulted in more up taking the drug. Therefore, it is possible to arise a question which if this mutation could increase the stability of the protein and followed by overexpression, then why the isolates had the phenotype of non-healing. The answer reflects the facts that molecular mechanisms of antimonial resistant are multifactorial (15).

Efllux of the drug (16) or inactivating the drugs or their metabolites are the other mechanisms that approved in the parasites (17). Although the isolates in our study showed overexpression of *LmAQP1*, after up taking the antimonial drugs, they may inactive or efflux from the parasite. Phosphorylation has not any effect on the channel activity but make stability (14). This stability makes amplified the other roles of the protein such as combating with osmotic stress. Based on knowledge, phosphorylation is dependent on oxidative stress presenting in environment, inside either the vector body or mammalian macrophages (14). Therefore, *LmAQP1* is phosphorylated during the metacyclic stage to provide stability. This helps to parasite to combat osmotic stress in metacyclic stage, the stage that is ready to infect the mammalian host.

The role of MAP2 for phosphorylation of threonine for providing stability of LmAQP1 and then overexpression needs more attention with the goal of developing a new approach for drug targeting. On the other hand, development of some compounds that target MAP2 for preventing phosphorylation and increasing the half-life of AQP1 may be dangerous because MAP kinases in all organisms are familiar and therefore they might target the ones from human, too (18). Although AQP1 protein belongs to the vast family of AQP in all organisms but finding some point nucleotides inside it might be the better target for designing the drug. This strategy for decreasing the stability of AQP1 effects on the other roles of this important protein such as combating the osmotic stress. Consequence, decreasing the power of parasite for exposing to osmotic stress might be a powerful tool for control of the disease. One of the approaches for combating the non-healing isolates was induction of MPK2 in order to increase the stability of AQP1 in the non-healing isolates to force them more up taking of Sb (III) (14). Our results are not agreed with the hypothesis of Mandal et al. (14) because the clinical non-healing isolates that we assessed in this study showed overexpression of *AQP1* but they did not respond to antimonial therapy.

The phenotypic characteristic in *Leishmania* for providing susceptibility or resistance to drugs is multifactorial and therefore more investigations are necessary to find the molecular mechanisms in detail by which *Leishmania* select. This is the first report of effective of G562A on *AQP1* gene expression in clinical non-healing isolates. Moreover, presenting a point mutation was reported inside AQP1 develops resistance isolate (7). G133D in AQP1 can be one of the mechanisms in non-healing isolates of *Leishmania*. Moreover, some other mutational analyses have been performed on LmAQP1 that revealed Ala163 and Glu152 are involved in metalloid uptake and reduced permeability to antimony (19, 20). Therefore, in this study, we discovered the new resistance mechanism corresponding to a point mutation of *AQP1*.

## Conclusion

The non-healing clinical isolates may have mutation of G562A in *AQP1*. This mutation can affect *LmAQP1* gene expression. The mutation of G562A resulted in providing additional threonine. Threonine may be the target of kinase enzyme for more stability of the AQP1 protein. Therefore, this alteration can be candidate for drug target.
